# Crystal structure of diamondback moth ryanodine receptor Repeat34 domain reveals insect-specific phosphorylation sites

**DOI:** 10.1186/s12915-019-0698-5

**Published:** 2019-10-09

**Authors:** Tong Xu, Zhiguang Yuchi

**Affiliations:** Tianjin Key Laboratory for Modern Drug Delivery & High-Efficiency, Collaborative Innovation Center of Chemical Science and Engineering, School of Pharmaceutical Science and Technology, Tianjin University, Tianjin, 300072 China

**Keywords:** Ryanodine receptor, Diamondback moth, Phosphorylation, Protein kinase A, Insecticide

## Abstract

**Background:**

Ryanodine receptor (RyR), a calcium-release channel located in the sarcoplasmic reticulum membrane of muscles, is the target of insecticides used against a wide range of agricultural pests. Mammalian RyRs have been shown to be under the regulatory control of several kinases and phosphatases, but little is known about the regulation of insect RyRs by phosphorylation.

**Results:**

Here we present the crystal structures of wild-type and phospho-mimetic RyR Repeat34 domain containing PKA phosphorylation sites from diamondback moth (DBM), a major lepidopteran pest of cruciferous vegetables. The structure has unique features, not seen in mammalian RyRs, including an additional α-helix near the phosphorylation loop. Using tandem mass spectrometry, we identify several PKA sites clustering in the phosphorylation loop and the newly identified α-helix. Bioinformatics analysis shows that this α-helix is only present in Lepidoptera, suggesting an insect-specific regulation. Interestingly, the specific phosphorylation pattern is temperature-dependent. The thermal stability of the DBM Repeat34 domain is significantly lower than that of the analogous domain in the three mammalian RyR isoforms, indicating a more dynamic domain structure that can be partially unfolded to facilitate the temperature-dependent phosphorylation. Docking the structure into the cryo-electron microscopy model of full-length RyR reveals that the interface between the Repeat34 and neighboring HD1 domain is more conserved than that of the phosphorylation loop region that might be involved in the interaction with SPRY3 domain. We also identify an insect-specific glycerol-binding pocket that could be potentially targeted by novel insecticides to fight the current resistance crisis.

**Conclusions:**

The crystal structures of the DBM Repeat34 domain reveals insect-specific temperature-dependent phosphorylation sites that may regulate insect ryanodine receptor function. It also reveals insect-specific structural features and a potential ligand-binding site that could be targeted in an effort to develop green pesticides with high species-specificity.

## Background

Ryanodine receptors (RyRs) are large ion channels located in the sarcoplasmic reticulum membrane, which control the calcium release from the intracellular store [1, 2]. They play a central role not only in excitation-contraction (EC) coupling of cardiac and skeletal muscles [3, 4], but also in learning and memory [5, 6]. Many mutations identified in RyRs have been associated with a number of muscular and neurological disorders, such as catecholaminergic polymorphic ventricular tachycardia (CPVT) [7, 8], arrhythmogenic right ventricular dysplasia (ARVD) [9], malignant hyperthermia (MH) [10–12], central core disease (CCD) [11, 13], and Alzheimer’s disease (AD) [14]. Three isoforms of RyRs have been identified in mammals: RyR1 is predominantly expressed in skeletal muscle, RyR2 is mainly found in cardiac muscle, and RyR3, first identified in the brain, is more ubiquitous [15–18].

By contrast, insects only have one type of RyR, which shares the highest sequence identity with RyR2 (~ 47%) [19]. As in mammals, insect RyRs are expressed in the muscular and central nervous systems [20]. Early studies by the pioneering groups on insect RyRs, such as in fruit flies, houseflies, and cockroaches, show that their activities can be regulated by caffeine, ATP, ryanodine, and its derivatives [3, 21–23]. Insect RyRs have drawn a lot of attention since the market introduction of diamide insecticides used to control a broad range of destructive agricultural pests. In fact, three diamide compounds (flubendiamide, chlorotraniliprole, and cyantraniliprole) generate worldwide annual sales of over 2 billion USD with a compound growth rate of over 50% [24]. Electrophysiological and biochemical studies have revealed the mode of action of diamide insecticides as activators of insect RyRs with a binding site different from ryanodine and caffeine [19, 25–27], but the exact binding site remains elusive. Recently, the widespread application of diamides has driven the evolution of resistance mutations due to the increased selection pressure [28–31]. Mutations found in the transmembrane helix two (S2) and helix four (S4) of RyR from diamondback moth (DBM), *Plutella xylostella*, (I4790M and G4946E, respectively), and the corresponding positions in tomato leafminer, *Tuta absoluta* (I4746M and G4903E), have induced over 1000-fold resistance against diamide insecticides [29, 32]. Due to the selection advantage, the resistant population has been spreading fast in many countries, including China, the USA, Japan, Korea, Thailand, Philippines, and Vietnam [33]. Hence, there is a pressing need for the development of novel insecticides that can control the resistant pests. Targeting a distinct binding site on these mutant RyRs is one potential solution.

The mammalian RyR is under control of cAMP-activated protein kinase A (PKA). During the fight-or-flight response, the activation of β-adrenergic receptor turns on adenylyl cyclase, which increases the intracellular levels of the second messenger cAMP and activates PKA [34, 35]. PKA phosphorylates several sites in RyRs, including S2843 in human RyR1, or S2030 and S2808 in RyR2 [36–39]. The phosphorylation increases the activity of the channel and causes the enhancement of skeletal muscle contraction and cardiac output [40]. However, the chronic activation of the β-adrenergic pathway causes hyperphosphorylation of RyR2, which is associated with heart failure [36, 41, 42]. Later study identified more phosphorylation sites in the same domain near the central region of the channel, namely Repeat34 domain, suggesting the importance of this domain in the phosphorylation regulation [43, 44].

In insects, the PKA pathway has not been well studied. In recent studies, Jing et al. show that the adipokinetic hormone 1 (AKH1) and the steroid hormone 20-hydroxyecdysone activate Gs-coupled Bombyx adipokinetic hormone receptor (AKHR) and ecdysone responsive G-protein-coupled receptors (ErGPCR1 and ErGPCR2), which in turn trigger an increase in the intracellular cAMP concentration and then activation of PKA. PKA enters the nucleus and phosphorylates cAMP response element-binding protein (CREB) to regulate gene expression [45, 46]. There are also some reports on the insect PKA phosphorylation of other substrates, including TG-lipase and lipid storage droplet protein 1 (Lsdp1) in Manduca sexta [47] and Broad-Complex transcription factor (BR-C) in silkworms [48]. To our knowledge, however, there is no detailed study on the PKA regulation on insect RyR. Nonetheless, the relatively high conservations of PKA and RyR proteins between mammals and insects suggest that the function of insect RyRs might also be regulated by PKA.

The crystal structures of Repeat34 domains (also called phosphorylation domains because they contain the major phosphorylation sites) from the mammalian RyRs have been solved previously [44]. In RyR1 and RyR2, the canonical phosphorylation sites are located in a flexible loop connecting two halves of the domain, but in RyR3, which cannot be phosphorylated, the flexible loop is replaced by a structured α-helix [44]. Several disease-causing mutations in this domain face the same side as the phosphorylation loop, suggesting that they might affect the same interface [43, 44]. The recent cryo-electron microscopy (cryo-EM) studies of full-length RyRs show that the RyR is a huge mushroom-shaped protein consisting of dozens of individual domains, and the Repeat34 domain is located at the turret region on the cytoplasmic side of the channel [49–54].

Here we present the atomic-resolution crystal structures of wild-type and phospho-mimetic RyR Repeat34 from DBM (*P. xylostella*), a destructive pest devouring cruciferous crops globally [55]. We identify several PKA-phosphorylation sites clustering in a small region of the domain by LC-MS/MS. Interestingly, the exact phosphorylation sites vary depending on the temperature of the reaction. DBM Repeat34 shows distinct structural elements only existing in Lepidoptera, which might contribute to the insect-specific phosphorylation regulation. Thermal melt experiment shows that the stability of DBM Repeat34 is significantly lower than the mammalian counterparts. Docking of DBM Repeat34 crystal structure into cryo-EM model of the full-length RyR reveals that the region containing phosphorylation sites shows very different conformation compared to mammalian RyR. Furthermore, the interface between Repeat34 and neighboring HD1 domain is more conserved compared to the phosphorylation loop region that might be involved in the interaction with SPRY domains or the voltage-gated calcium channel. We identify a glycerol-binding pocket in DBM Repeat34 surrounded by several positively charged residues that are not present in mammalian RyRs. This unique binding pocket might serve as an insect-specific druggable pocket for developing novel insecticides that target resistant pests.

## Results

### The crystal structures of DBM Repeat34 domain

**Fig. 1:**
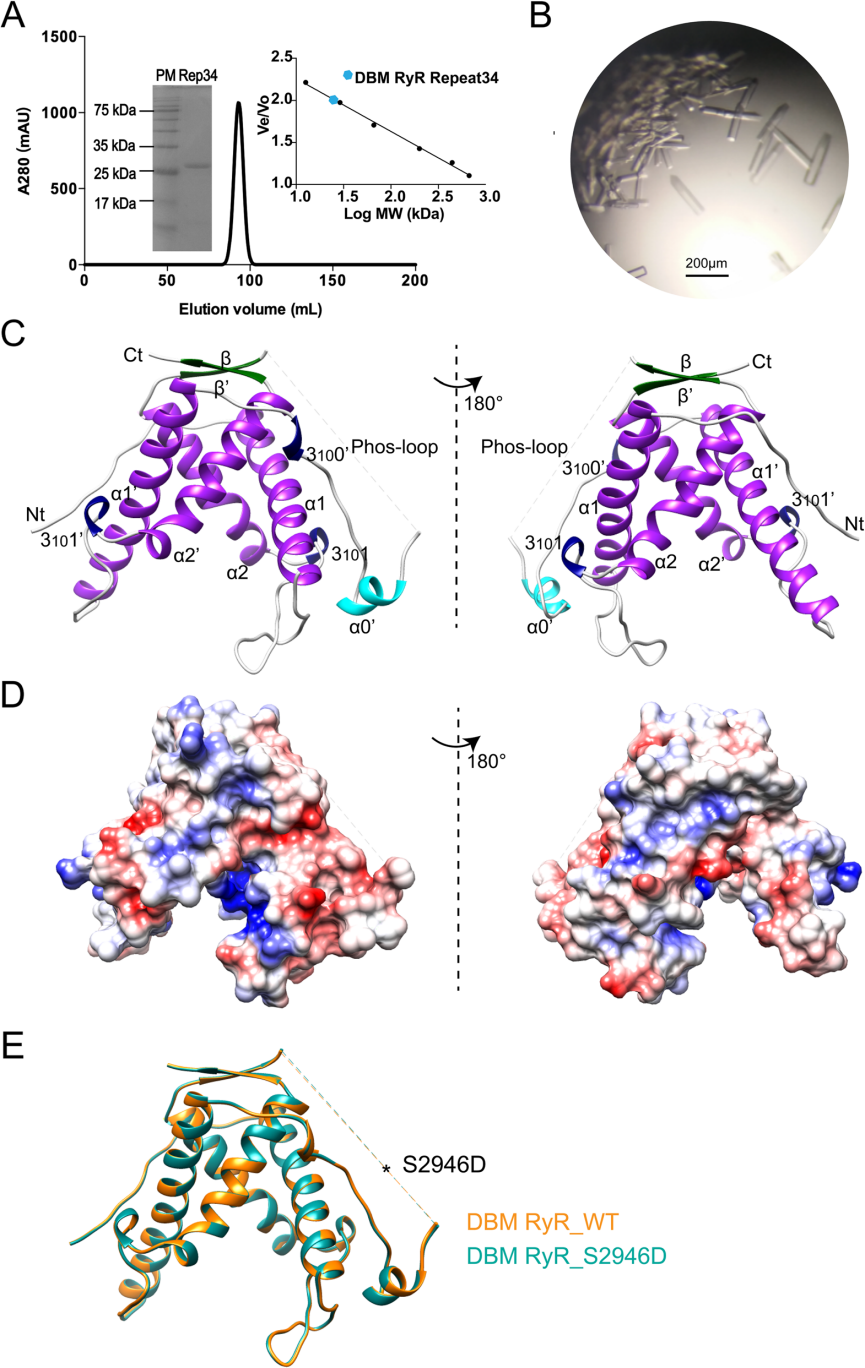
Structure of DBM Repeat34**. a** Elution profile of DBM RyR Repeat34 by gel-filtration chromatography using a Superdex 200 26/600 column (GE Healthcare, USA). The right inset shows the plotted standard curve for this column. The molecular weight estimated from its elution volume is ~ 28.1 kDa, suggesting monomeric form in solution (predicted MW is 24.9 kDa). The left inset is a 15% SDS-PAGE of purified DBM RyR Repeat34 showing protein marker (PM) in the left lane and purified Repeat34 (Rep34) in the right lane. **b** DBM RyR Repeat34 crystals produced by the hanging-drop method. **c** The crystal structure of DBM RyR Repeat34 in two different views are colored according to their secondary structure elements: α helixes in purple, 3_10_ helixes in blue, β strands in green, and loops in gray. The Lepidoptera-specific helix α0′ is highlighted in cyan. **d** Electrostatic surface views of DBM RyR Repeat34. Negatively charged, positively charged, and non-charged surfaces are colored in red, blue, and white, respectively. **e** Structural superposition of the wild type and the S2946D mutant of DBM RyR Repeat34. The phospho-mimetic mutation is located in a flexible loop shown as dash line

**Fig. 2:**
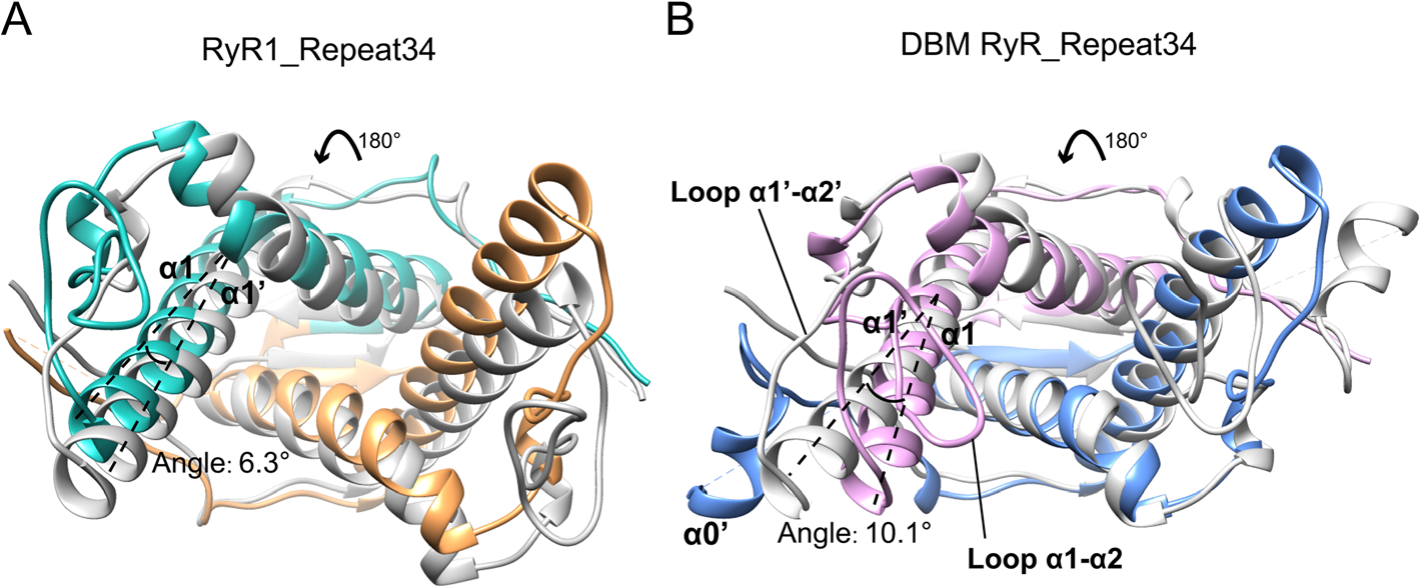
Twofold symmetry. **a** Superposition of RyR1 Repeat34 domain onto itself via a 180° rotation shows that the domain has a clear intrinsic twofold symmetry. Its original N-terminal region and C-terminal region are displayed in cyan and orange, respectively, and the superposed version is displayed in gray. **b** Superposition of DBM RyR Repeat34 domain onto itself via a 180° rotation shows that the intrinsic twofold symmetry is compromised by the presence of helix α0′, the difference between helix α1 and helix α1′, and the difference between loop α1-α2 and loop α1′-α2′. Its original N-terminal region and C-terminal region are displayed in pink and blue, respectively, and the superposed version is displayed in gray

DBM Repeat34 domain (residues 2836–3050) was recombinantly expressed in *E. coli* and purified to homogeneity using a five-step purification protocol. It showed as a monomer in solution and was crystallized as rod-shaped crystals (Fig. 1a, b). We could not solve the phase problem using either mammalian Repeat12 or Repeat34 structures as template due to the significant structural divergence among them. After extensive trials, we solved the crystal structure of wild-type DBM Repeat34 at 1.85 Å using a combined search model consisting of the second half of RyR2 Repeat34 (PDB 4ETV) and the first half of RyR1 Repeat12 (PDB 5C30) as molecular replacement template (Table 1). There is only one molecule in the asymmetric unit (ASU). DBM Repeat34 is a horseshoe-shaped domain consisting of five α-helices, two β-strands, and three 310-helices (Fig. 1c). It has a clearly intrinsic twofold symmetry as previously reported for RyR1 Repeat34 [44], but the symmetry is partially broken by the extra helix α0′ that is only present in the second half of the domain, the difference between helices α1 and α1′, and the difference between loops α1-α2 and α1′-α2′ (Fig. 2). The two halves of DBM Repeat34 is connected by a long flexible loop previously defined as “phosphorylation loop,” where the classic phosphorylation sites are located (Fig. 1c). Several phosphorylation sites in DBM RyR that are newly identified in this study also cluster in this loop (described later). The surface of the domain is mainly negatively charged with a small positively charged pocket located in the inner side of the horseshoe formed by helices α1 and α2 (Fig. 1d). We also solved the crystal structure of S2946D, a phospho-mimetic mutant of DBM Repeat34 which mimics a permanent homogenous phosphorylation state, at 1.53 Å resolution (Table 1). The mutation is located in the middle of the flexible phosphorylation loop and does not change the structure of the domain significantly (Fig. 1e). The overall root mean square deviation (RMSD) between the wild-type (WT) and mutant structures is 0.17 Å for 192 Cα atoms (residues 2838–2930 and 2952–3050 for both WT and mutant).

**Table 1:** Data collection and refinement statistics

Crystal	Repeat34 wild type	Repeat34 S2946D
*λ* for data collection (Å)	0.9795	0.9795
Data collection		
Space group	P 31 2 1	P 31 2 1
Cell dimension (Å)		
a, b, c (Å)	55.99|55.99|132.59	55.82|55.82|131.55
α, β, γ, (°)	90.00, 90.00, 120.00	90.00, 90.00, 120.00
Resolution	28–1.85 (1.91–1.85)	27.91–1.53 (1.58–1.53)
Rmerge†	0.068 (0.715)	0.109 (0.507)
Average I/σ(I)	25.75 (2.5)	27.88 (2.07)
Completeness (%)	99.85 (99.04)	99.61 (96.28)
Redundancy	9.6 (9.9)	8.3 (4.6)
*Z*	1	1
Refinement		
Resolution	28–1.85 Å	27.91–1.53 Å
No. of reflections	21,341	36,581
*R*_factor_/*R*_free_ (10% data)	0.186/0.234	0.190/0.225
RMSD length (Å)	0.012	0.020
RMSD angle (°)	1.04	1.55
No. of atom		
Protein	1762	1851
Ligands	22	65
Water	179	214
Ramachandran plot (%)		
Most favored	98.94	99.47
Additionally allowed	1.06	0.53

Values in parentheses refer to the highest resolution shell

### DBM RyR Repeat34 domain versus mammalian RyR Repeat domains

**Fig. 3:**
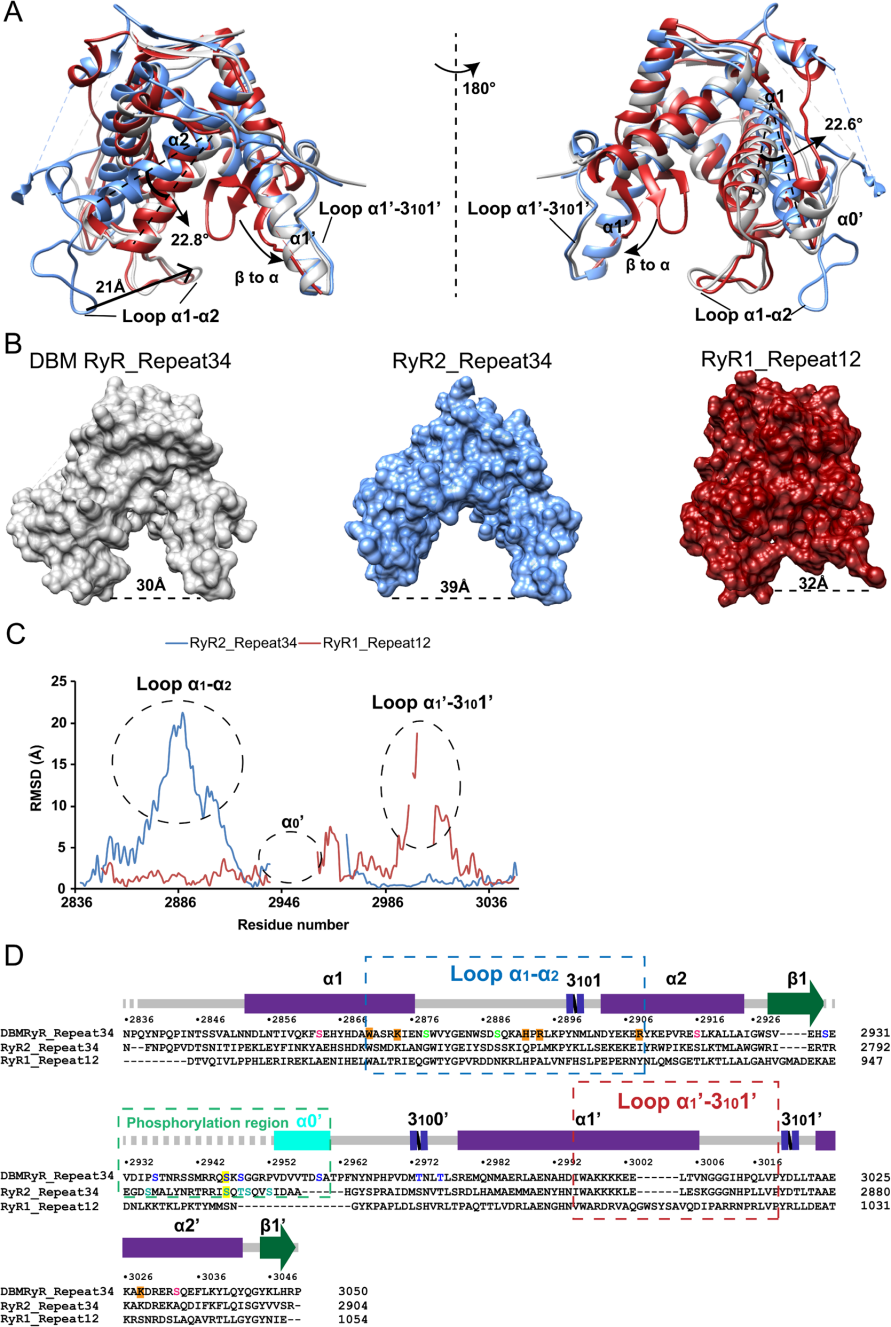
Comparison of repeat domains. **a** Superpositions of the DBM RyR Repeat34 crystal structure (gray) with mouse RyR2 Repeat34 crystal structure (blue; PDB ID 4ETV) and rabbit RyR1 Repeat12 crystal structure (red; PDB ID 5C30). The main structural differences are labeled. **b** Comparison of the surface views of the three structures in panel A. The distance between the two ends of each horseshoe is labeled. **c** Plot showing per residue root mean square deviation (RMSD) for the mouse RyR2 Repeat34 and rabbit RyR1 Repeat12 crystal structures relative to DBM RyR Repeat34 crystal structure. **d** Sequence alignment of DBM Repeat34, mouse RyR2 Repeat34, and rabbit RyR1 Repeat12. Secondary structure elements for DBM RyR Repeat34 are indicated above the sequence. Phosphorylation sites in DBM are colored in blue, green, and pink depending on their location within the 3-dimensional structure of the domain (same color scheme as in Fig. 6). Phosphorylation sites in RyR2 are colored in turquoise. The classic PKA site RyR2 S2808 and the equivalent PKA site DBM RyR S2946 are highlighted in yellow. Residues contributing to the glycerol binding are highlighted in orange

**Fig. 4:**
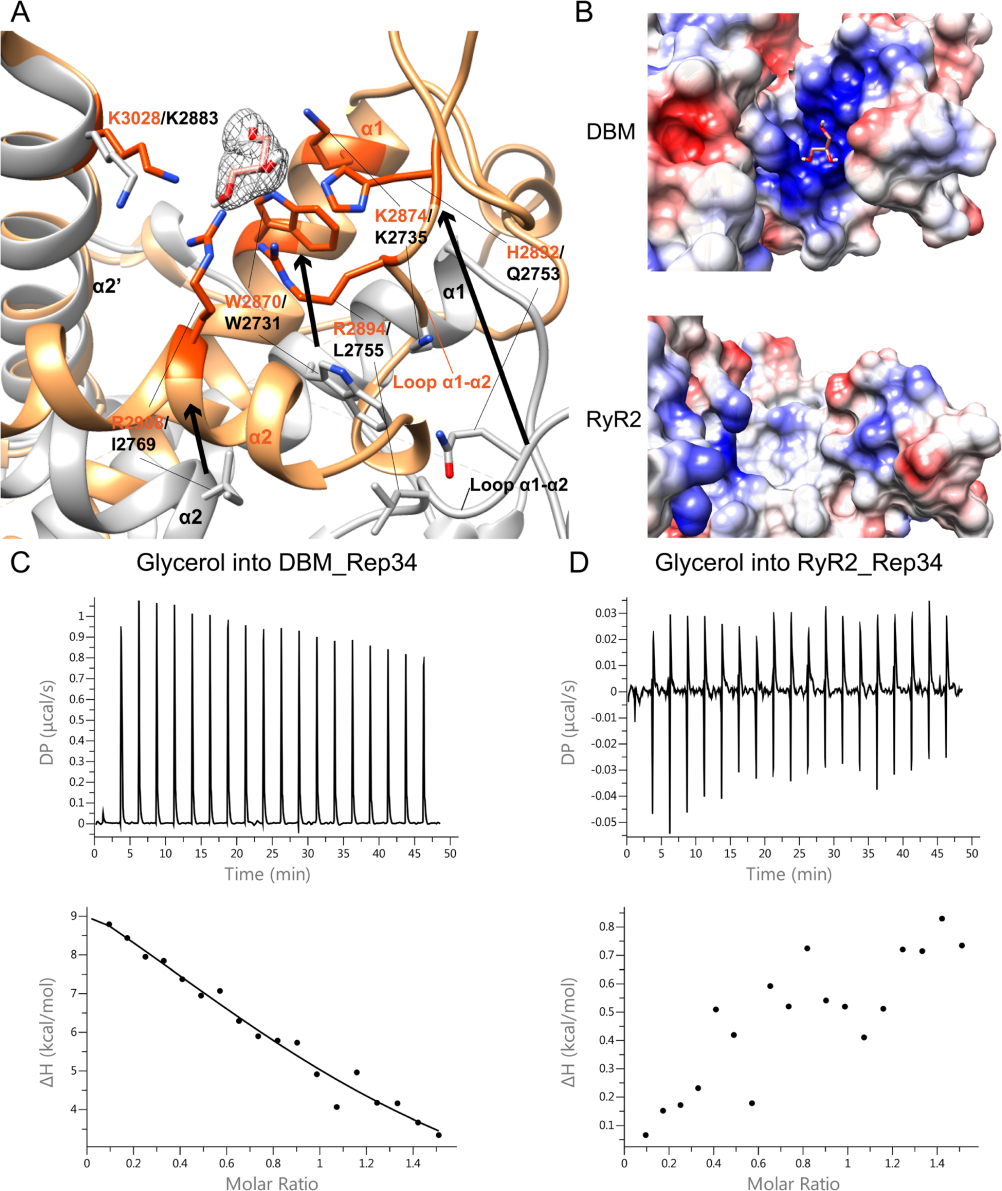
Insect-specific binding pocket. **a** Glycerol-binding pocket in DBM RyR Repeat34 is shown in orange. The side chains of the substrate-interacting residues are labeled and displayed in dark orange. The 2mFo-DFc electron density map of glycerol is depicted in mesh and displayed at 1σ. The same region of RyR2 Repeat34 is shown in white for comparison. **b** Electronic surface view of glycerol-binding pocket in DBM RyR Repeat34 shows a positively charged surface. Electronic surface view of the same region in RyR2 Repeat34 shows a non-charged surface. **c** ITC binding isotherms show the interaction between glycerol (200 μM as titrant) and DBM RyR Repeat34 (25 μM in the cell) and the measured Kd is 17.7 μM. **d** ITC binding isotherms show no clear interaction between glycerol (200 μM as titrant) and mouse RyR Repeat34 (25 μM in the cell)

The sequence identity between DBM Repeat34 and mammalian RyR1, RyR2, and RyR3 are 43.5%, 46.1%, and 44.1%, respectively. Despite the relatively high sequence identity, there is surprisingly a significant structural variation between DBM Repeat34 and mammalian Repeat34 domains. The RMSD between superposed DBM RyR and RyR1, RyR2, and RyR3 are 5.42 Å for 181 Cα atoms (DBM 2838–2930, 2952–2954, and 2965–3049; RyR1 2734–2829 and 2855–2939), 9.61 Å for 187 Cα atoms (DBM 2839–2930, 2952–2954, and 2958–3049; RyR2 2700–2801 and 2820–2904), and 7.97 Å for 188 Cα atoms (DBM 2839–2930, 2952, 2954–2963, and 2965–3049; RyR3 2598–2690, 2692, 2699–2707, and 2716–2800), respectively. There are three main differences between DBM Repeat34 and mammalian Repeat34 domains. First, α1 and α2 helices and α1-α2 loop from the first half of the domain adopt very different conformations (Fig. 3a). Comparing DBM Repeat34 with RyR2 Repeat34, the angles between two α1 and two α2 helices are 22.6° and 22.8°, respectively, and the centers of the two α1-α2 loops are ~ 21 Å apart (measured by the structural analysis tool in UCSF chimera [56] (Fig. 3a, c). Second, DBM Repeat34 has an extra helix α0′ that is not observed for all three mammalian Repeat34 domains. This peculiar helix α0′ is right in the middle of the phosphorylation loop and behind the flexible portion (Fig. 3a, c, d). Tandem mass spectrometry has located a new phosphorylation site within this helix (described later). Third, there is one glycerol molecule trapped in a positively charged pocket formed by residues from helix α1, helix α2, helix α2’, and loop α1-α2, including Trp2870, Lys2874, His2892, Arg2894, Arg2908, and Lys3028 (Fig. 4a). In RyR2, the equivalent residues for Arg2894 and Arg2908 are Leu2755 and Ile2769, respectively, which have higher hydrophobicity (Fig. 4b, c). In DBM RyR, helix α1 and helix α2 move towards helix α2′ to close down the distance between the two halves of the domain. Meanwhile, loop α1-α2 folds back to act like a lid to partially close the opening of the horseshoe (Fig. 4a). As a result, the distance between two ends of horseshoe is narrowed down by ~ 9 Å in DBM RyR compared to RyR2 (Fig. 3b). This new ligand-binding pocket is near the bottom of the horseshoe, formed by an intra-domain clamshell movement of DBM RyR relative to RyR2. To prove the validity of this glycerol-binding pocket, we performed an ITC experiment to titrate glycerol into purified DBM Repeat34, which showed a clear binding with a Kd of 18 μM (Fig. 4c). In contrast, no binding could be detected between glycerol and RyR2 Repeat34 (Fig. 4d).

There is another homologous domain of Repeat34 called Repeat12 domain in RyR, spanning residues 861–1054, which probably has evolved by gene duplication. The crystal structure of RyR1 Repeat12 has been solved previously and shows structural similarity with Repeat34 domains [57]. Surprisingly, we found that the phosphorylation loop and the first half of DBM Repeat34 domain are more similar to RyR1 Repeat12 (RMSD 1.49 Å for 82 Cα atoms, residues 2849–2930) than mammalian Repeat34 (RMSD 7.14 Å for 92 Cα atoms, residues 2839–2930) (Fig. 3a, c). This explains why the initial effort to use mammalian Repeat34 domains as search model for molecular replacement had failed. The main difference between DBM Repeat34 and RyR1 Repeat12 lies in the second halves of the domains: the equivalent position of α1′ helix in DBM Repeat34 is a 3-strand β sheet in RyR1 Repeat12.

### Thermal stability

**Fig. 5:**
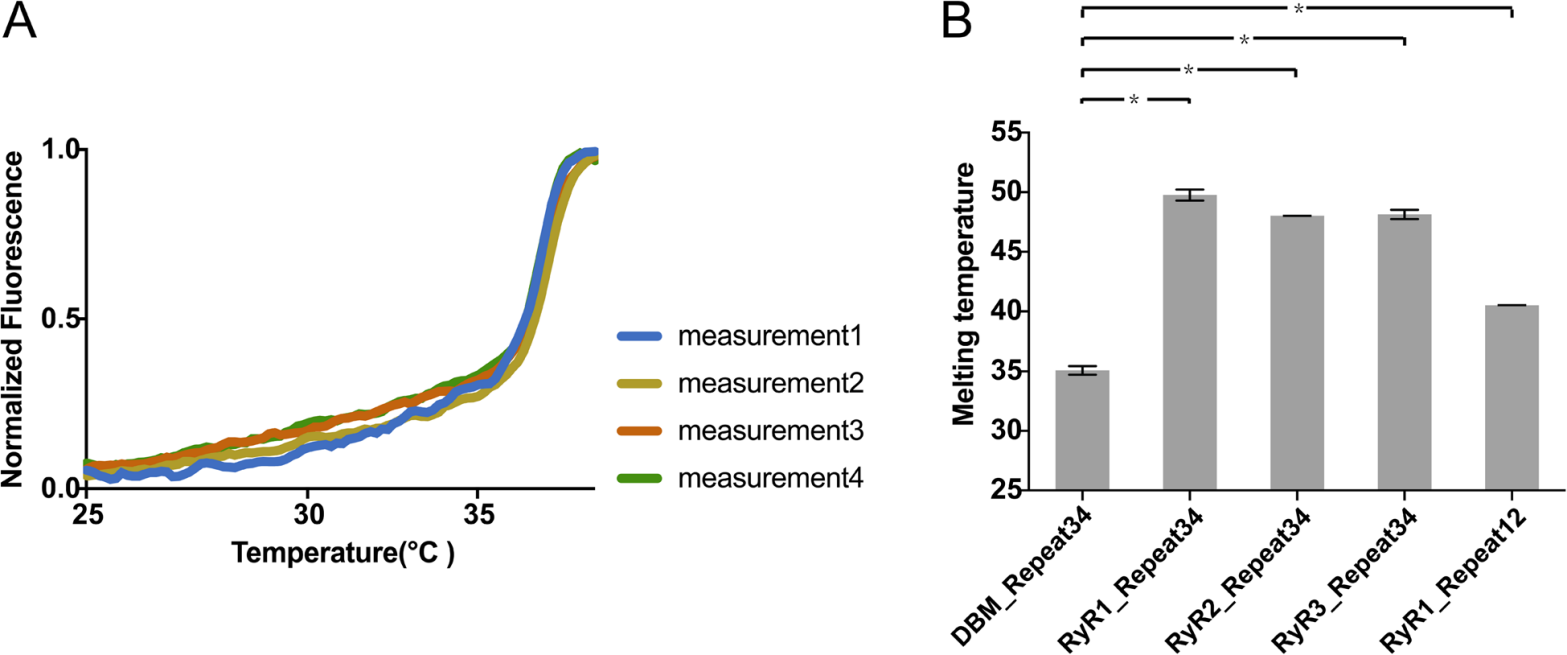
Thermal melt analysis. **a** Four measurements of thermal melt curves for DBM RyR Repeat34. **b** Comparison of the melting temperatures for Repeat34 from DBM RyR, rabbit RyR1, mouse RyR2, human RyR3, and Repeat12 from rabbit RyR1. Error bars show the standard deviation. *P < 0.0001 (two-tail Student *T* test)

We probed the thermal stability of DBM RyR Repeat34 by the fluorescence-based thermal shift assay. Its melting temperature (Tm) (35.0 °C) is significantly lower than the Tm of RyR1 Repeat34 (49.8 °C), RyR2 Repeat34 (48.0 °C), RyR3 Repeat34 (48.1 °C), and RyR1 Repeat12 (41.1 °C) [44, 57] (Fig. 5a, b). The low Tm of DBM Repeat34 agrees with the observation that the protein easily aggregates at room temperature during the purification process. Previously, it was reported that a single RyR1 mutation L2867G that causes malignant hyperthermia (MH) can reduce the Tm of RyR1 Repeat34 by 13 °C [44]. One common mechanism for MH or CPVT mutations is to affect the stability and cause misfolding of RyR domains, consequently causing the gain-of-function phenotype of the channel [58]. Thus, some non-conserved residues in DBM Repeat34 could cause the destabilization of the domain, which might contribute to the high activity of the channel [59]. Unlike mammalian RyRs that normally function at 37 °C, insects live at relatively lower ambient temperature. This might promote the reduction of thermal stability of DBM RyR domains through evolution.

### PKA phosphorylation sites in DBM RyR

Previous studies showed that Ser2843 in RyR1 and the equivalent residue Ser2808 in RyR2 from their Repeat34 domains could be phosphorylated by PKA [36, 37, 44]. This Ser is conserved in DBM RyR (Fig. 3d). There is an additional calmodulin-dependent kinase II (CaMKII) site Ser2814 in RyR2 [60], which is replaced by Pro2952 in DBM RyR (Fig. 3d). To determine the specific PKA phosphorylation sites on DBM RyR, we phosphorylated DBM Repeat34 domain in vitro and used tandem mass spectrometry to identify the phosphorylated residues.

**Fig. 6:**
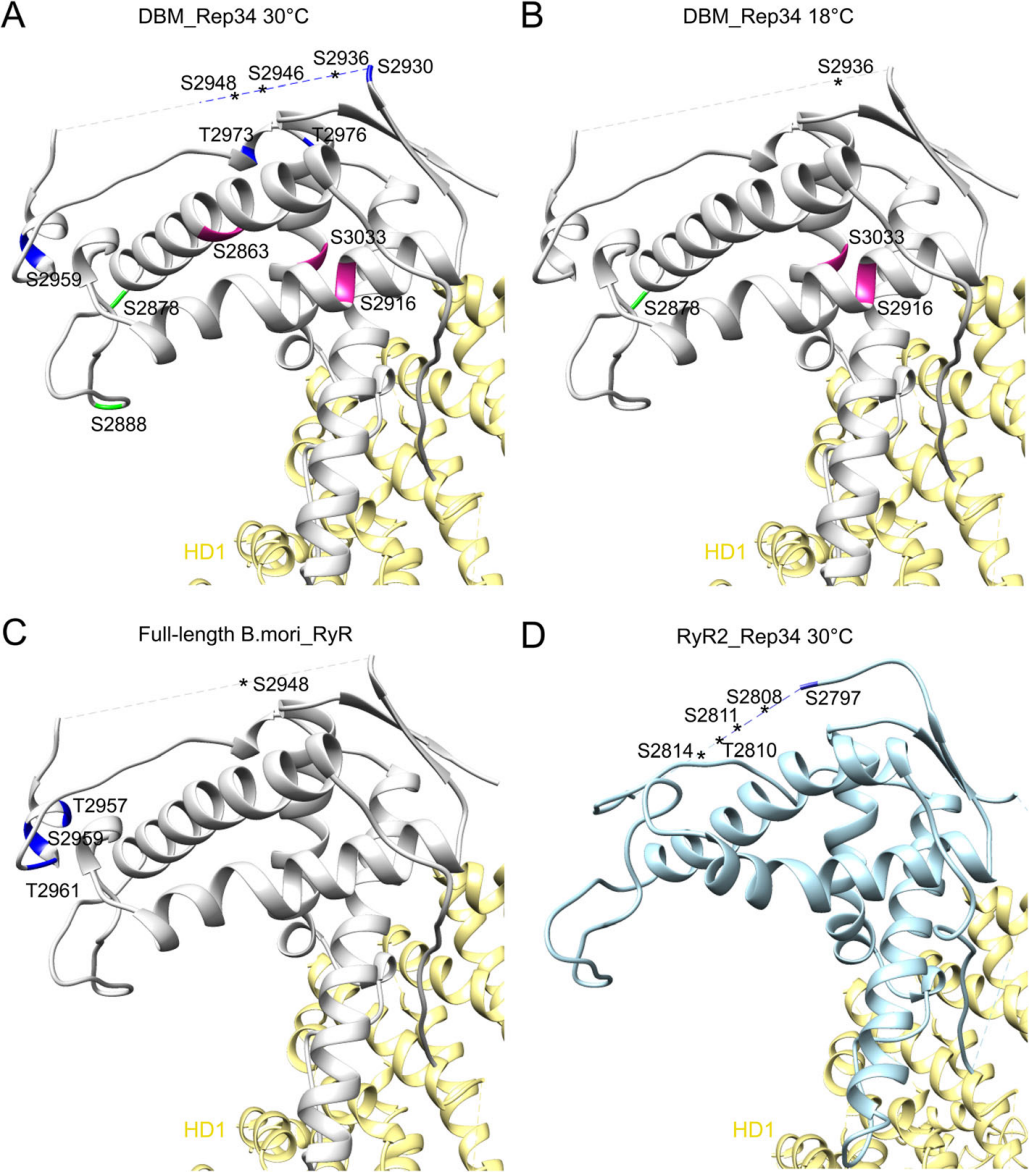
Temperature-dependent Phosphorylation. **a** High-temperature phosphorylation sites of DBM RyR Repeat34 measured by LC-MS/MS. The buried sites are colored in pink, the sites located in the phosphorylation loop are colored in blue, and the other sites are colored in green. RyR2 HD1 domain is shown in yellow. **b** Low-temperature phosphorylation sites of DBM RyR Repeat34 measured by LC-MS/MS. The color scheme is the same as in panel A. **c** Phosphorylation sites of full-length RyR from silkworm measured by LC-MS/MS. The color scheme is the same as panel A. **d** High-temperature phosphorylation sites of mouse RyR2 Repeat34 measured by LC-MS/MS

We first performed the phosphorylation assay at 30 °C. There are a total of 12 PKA sites identified in DBM Repeat34, which can be classified into three groups (Table 2, Fig. 6a). The first group contains seven sites clustered in the phosphorylation loop, including one in helix α0′ (S2959), four in the flexible region (S2930, S2936, S2946, S2948), and two in the structured loop region (T2973, T2976). The second group contains two sites (S2878, S2888) located in the core of Repeat34 but facing the same surface as the phosphorylation loop. The third group includes three residues (S2863, S2916, S3033), which are not accessible to the solvent in the presence of the neighboring domains. Thus, the phosphorylation of the third group might be an artifact due to the isolation of Repeat34 domain in the recombinant expression system or strong structural dynamics of the region.

**Table 2:** Detected phosphopeptides at 30 °C and 18 °C reactions, with each phosphorylated residue marked with star

Sequence	Theo.MH+ [Da]	DeltaM [ppm]	Site1	Site2	Site3
30 °C					
FS*EHYHDAWASR	1585.62198	− 0.15	2863		
IENS*WVYGENWSDSQK	2021.82768	6.01	2878		
KIENS*WVYGENWSDSQK	2149.92264	0.14	2878		
KIENS*WVYGENWSDS*QKAHPR	2611.17254	− 1.34	2888		
YKEPVRES*LK	1328.66099	0.88	2916		
EPVRES*LK	1037.5027	0.5	2916		
ALLAIGWSVEHS*EVDIPSTNR	2374.14387	2.47	2930		
ALLAIGWSVEHSEVDIPS*TNRSSMR	2835.34952	5.99	2936		
RQS*KSGGRPVDVVTDSATPFNYNPHPVDMTNLTLSR	4052.92842	1.35	2946		
S*GGRPVDVVTDSATPFNYNPHPVDMTNLTLSR	3553.64174	10.58	2948		
SGGRPVDVVTDS*ATPFNYNPHPVDMT*NLT*LSR	3713.5744	12.04	2959	2973	2976
SGGRPVDVVTDSATPFNYNPHPVDMT*NLTLSR	3537.64682	9.43	2973		
S*QEFLK	831.36481	− 0.65	3033		
DRERS*QEFLK	1387.63657	− 0.1	3033		
ERS*QEFLK	1116.50852	0.42	3033		
18 °C					
KIENS*WVYGENWSDSQK	2149.92264	− 0.2	2878		
ERS*QEFLK	1116.50852	1.07	2916		
ALLAIGWSVEHSEVDIPS*TNRSSMRR	3007.44554	1.03	2936		
YKEPVRES*LK	1328.66099	− 0.45	3033

Typically, the phosphorylation by PKA requires the substrate to be in an unfolded state. For example, phospholamban unfolds from a helix to an extended loop during PKA phosphorylation [61]. In the previous study, all the phosphorylation sites identified in RyR1 and RyR2 are in the loop regions [44]. In RyR3, which cannot be phosphorylated, the phosphorylation loop is replaced by a structured α helix [44]. The phosphorylation reactions for DBM and mammalian Repeat34 domains were all carried out at 30 °C, and we observed more phosphorylation modifications in DBM RyR, several of which are located in the region with secondary structures. Since the Tm of DBM Repeat34 is significantly lower than that of mammalian Repeat34, we suspect that at 30 °C some secondary structure elements in the domain are partially unfolded, which might change the phosphorylation pattern. According to the melting curve, 16% of the domain should be unfolded at 30 °C (Fig. 5). To test the hypothesis that the PKA phosphorylation of DBM Repeat34 is temperature-dependent, we repeated the in vitro phosphorylation assay at 18 °C. This time, we only identified four phosphorylation sites, including S2936 from the first group, S2878 from the second group, and S2916 and S3033 from the third group, proving the other eight phosphorylation sites are in fact temperature-dependent (Fig. 6b). These eight phosphorylation sites are either in the middle of secondary structures or at the interface with structured regions, which would hinder the access of PKA.

**Fig. 7:**
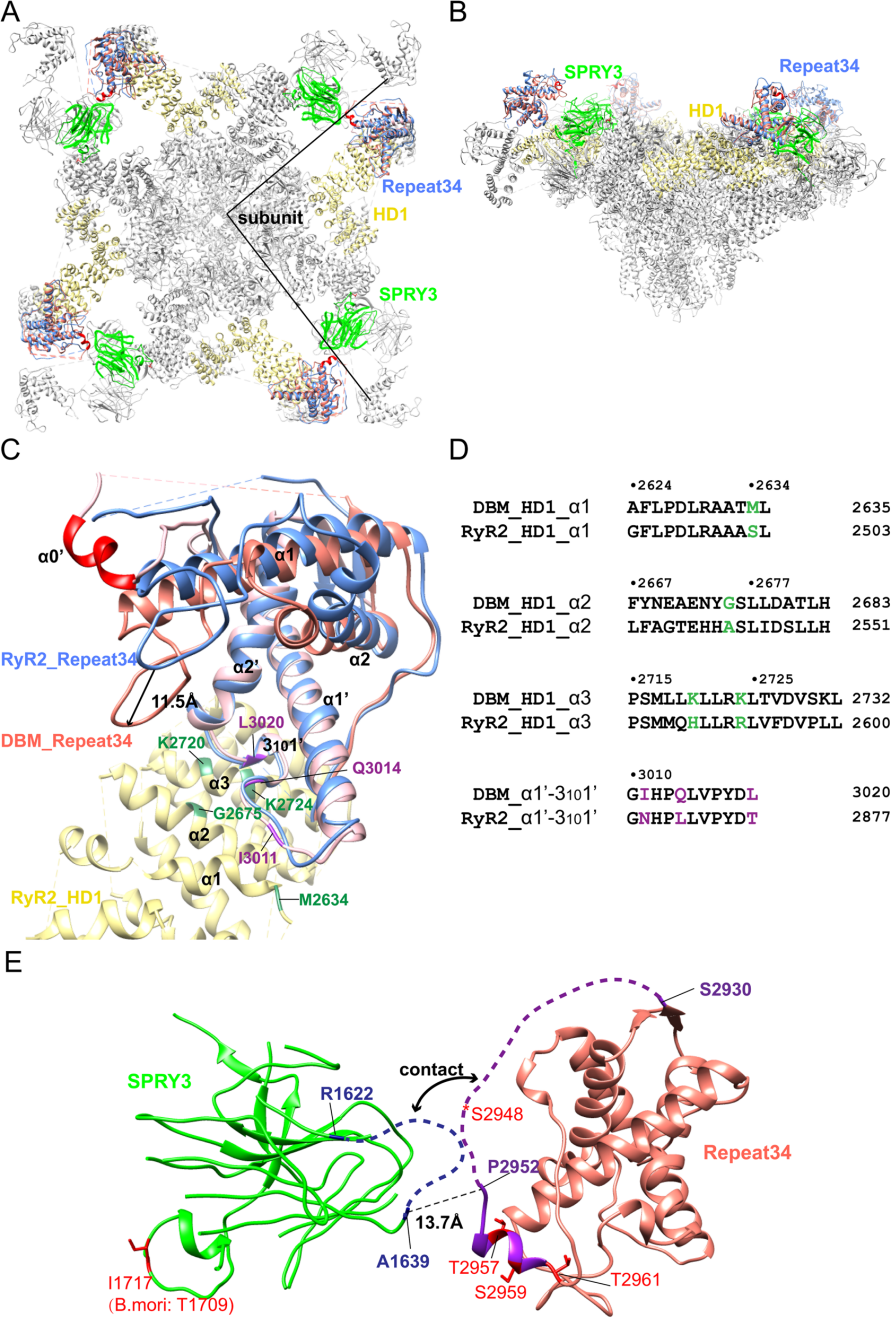
Position in Full-length RyR. **a** Top view of the position of DBM RyR Repeat34 superposed onto the RyR2 cryo-EM model (PDB ID 5GO9). DBM RyR Repeat34, RyR2 Repeat34, RyR2 HD1, and RyR2 SPRY3 are colored in pink, blue, yellow, and green, respectively. Helix α0′ is highlighted in red. The boundaries of a single subunit are indicated by straight lines. **b** Side view of the docked DBM RyR Repeat34. **c** Zoomed-in view of the docked DBM RyR Repeat34 (pink). RyR2 HD1 and Repeat34 domains are colored in yellow and blue, respectively. The region interacting with HD1 is more structurally conserved than the phosphorylation loop. The DBM-specific residues in Repeat34-HD1 interfaces are colored in purple (Repeat34) and green (HD1). **d** Sequence alignment between DBM RyR and pig RyR2 for the HD1-Repeat34 interface region, with the main residues involved in the contact colored in green and purple, respectively. **e** Zoomed-in view of the interface between DBM RyR Repeat34 and the neighboring SPRY3 domain. The two loops involved in the potential contact are shown in dash. Identified phosphorylation sites from full-length insect RyR are colored in red

To validate the phosphorylation sites identified from the in vitro assay and investigate the phosphorylation regulation under physiological condition, we purified the sarcoplasmic reticulum (SR) membrane from silkworm and used tandem mass spectrometry to identify the phosphorylated residues in the native full-length RyR. Silkworm and DBM share high similarity in RyR protein sequence. Totally, five potential phosphorylation sites were identified. Four (S2948, T2957, S2959 and T2961) are in the Repeat34 domain (Figs. 6c and 7e) and one is in SPRY3 domain (I1717) (Fig. 7e). All four phosphorylation sites in Repeat34 are conserved between DBM and silkworm. The site in SPRY3 domain is a Thr in silkworm but an Ile in DBM. Mass spec result showed that there was only one residue that was phosphorylated in this peptide containing four potential phosphorylation sites (Table 3). Considering that two sites (T2957 and T2961) were not detected in the experiment using isolated Repeat34 domain, we propose that likely either S2948 from the phosphorylation loop or S2959 from the helix α0′ is phosphorylated under the physiological condition.

**Table 3:** Detected phosphopeptides in native silkworm, with each potential phosphorylated residue marked with star

Sequence	Theo.MH+ [Da]	DeltaM [ppm]	Site1
SPTSLPLSAAVLPTSDKHVT*PQFPPRLK	3064.6231	0.58	1717
QSKS*GGEEEVQIEVSDTATRPVDVVT*DS*AT*PFNYNPHPVDMTNLTLSR	5327.46859	0.19	2948/2957/2959/2961

We also compared the phosphorylation sites identified from RyR2 Repeat34 with the ones from DBM Repeat34 (Figs. 3d and 6d). Two sites in the phosphorylation loop (S2946 and S2948) are conserved in RyR2 Repeat34. And there are also several other phosphorylation sites clustering in the phosphorylation loops in both domains, suggesting the mechanism of phosphorylation regulation on the hotspot loop is relatively conserved among species.

### Location within full-length RyR

Since the recent “resolution revolution” of cryo-EM technology, several cryo-EM structures of mammalian RyRs in high details have been solved, revealing the domain organization of full-length RyRs [49–54]. The Repeat34 domain is located at the top turret region of RyR projecting away from the main body of its cytoplasmic cap (Fig. 7a, b). We placed the crystal structure of DBM Repeat34 into the cryo-EM model of RyR2 (pdb: 5GO9) by selectively superposing two Repeat34 domains (Fig. 7c). Interestingly, we found that although the sequence identity of the first half (residues 2853–2929) and second half (residues 2979–3048) of Repeat34 domain-pair is both high (46.3% and 62.9%, respectively), structurally, the second half of the domain, which interacts with the neighboring HD1 domain, is much more conserved (RMSD 1.0 compared to 7.7 for the first half) (Fig. 7c). Similarly, the surfaces of HD1 in contact with Repeat34 are also relatively conserved (Fig. 7d). Several residues in the interface of these two domains are DBM-specific, including Ile3011, Gln3014, and Leu3020 in Repeat34 domain and Met2634, Gly2675, Lys2720, and Lys2724 in HD1 domain. Interestingly, these two groups of residues interact with each other, implicating the species-specific regulation developed through co-evolution. By contrast, the first half of DBM Repeat34 adopts a very different conformation in the pseudo-atomic model compared to RyR2. Its whole phosphorylation loop moves ~ 11.5 Å towards HD1 domain, and the unique helix 0’ pulls the phosphorylation loop towards the central pore region of the channel (Fig. 7c). Overall, the phosphorylation hot-spot region is more divergent compared to the region involved in HD1 interactions. On the other side of the domain, the phosphorylation loop and helix α0′ from Repeat34 are in close contact with a loop from SPRY3 in the full-length RyRs (Fig. 7a, b, e), implying that the phosphorylation might affect the interaction between these two domains.

**Fig. 8:**
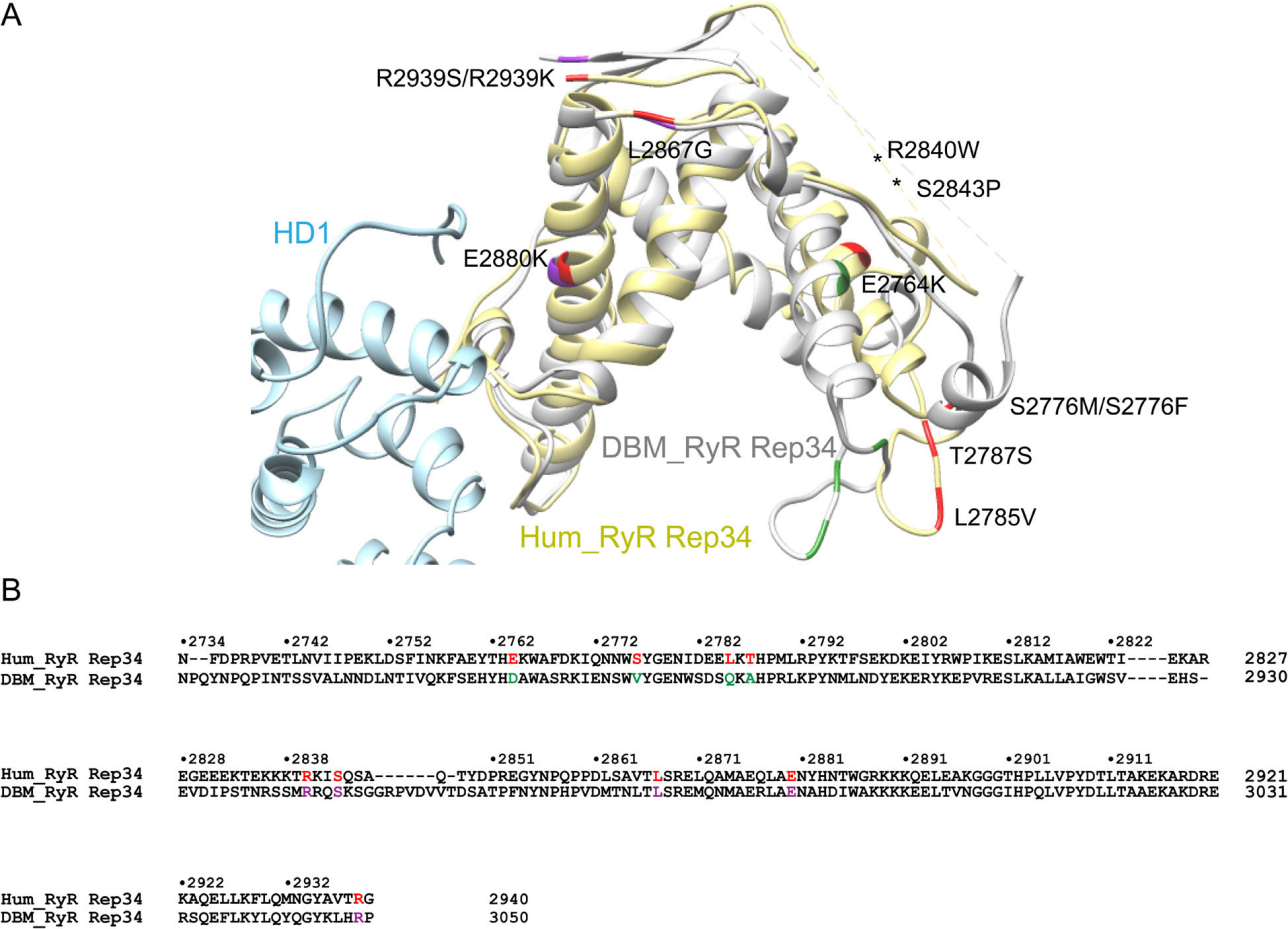
Disease-associated mutations mapped onto DBM Repeat34. **a** The crystal structure of DBM RyR Repeat34 is superposed with RyR1 cryo-EM model (PDB ID 5TAQ). Disease-associated mutations are colored in red in RyR1 structure. Corresponding conserved and non-conserved residues in DBM Repeat34 are colored in purple and green, respectively. **b** Sequence alignment between human RyR1 Repeat34 and DBM RyR Repeat34. The color scheme for disease mutations is the same as in panel A

There are several mutations identified in human RyR1 Repeat34 that are associated with MH or CCD diseases. We mapped these mutations on DBM Repeat34 and analyzed their sequence conservation. Interestingly, we found that the mutations in the first half of Repeat34 (E2764K, S2776 M/F, L2785 V, T2787S), which shows higher structural divergence, are at the positions that have non-conserved residues. In contrast, the mutations in the second half of the domain (L2867G, E2880K, R2840W, S2843P, R2939S/K), which is in contact with HD1 domain and structurally more conserved, are all at the positions that have conserved residues (Fig. 8a, b). This hints that the first half of the domain might be more tolerable to the variation of protein sequence and the mutations in this region might have relatively milder phenotypes.

### Phylogenetic analysis

**Fig. 9:**
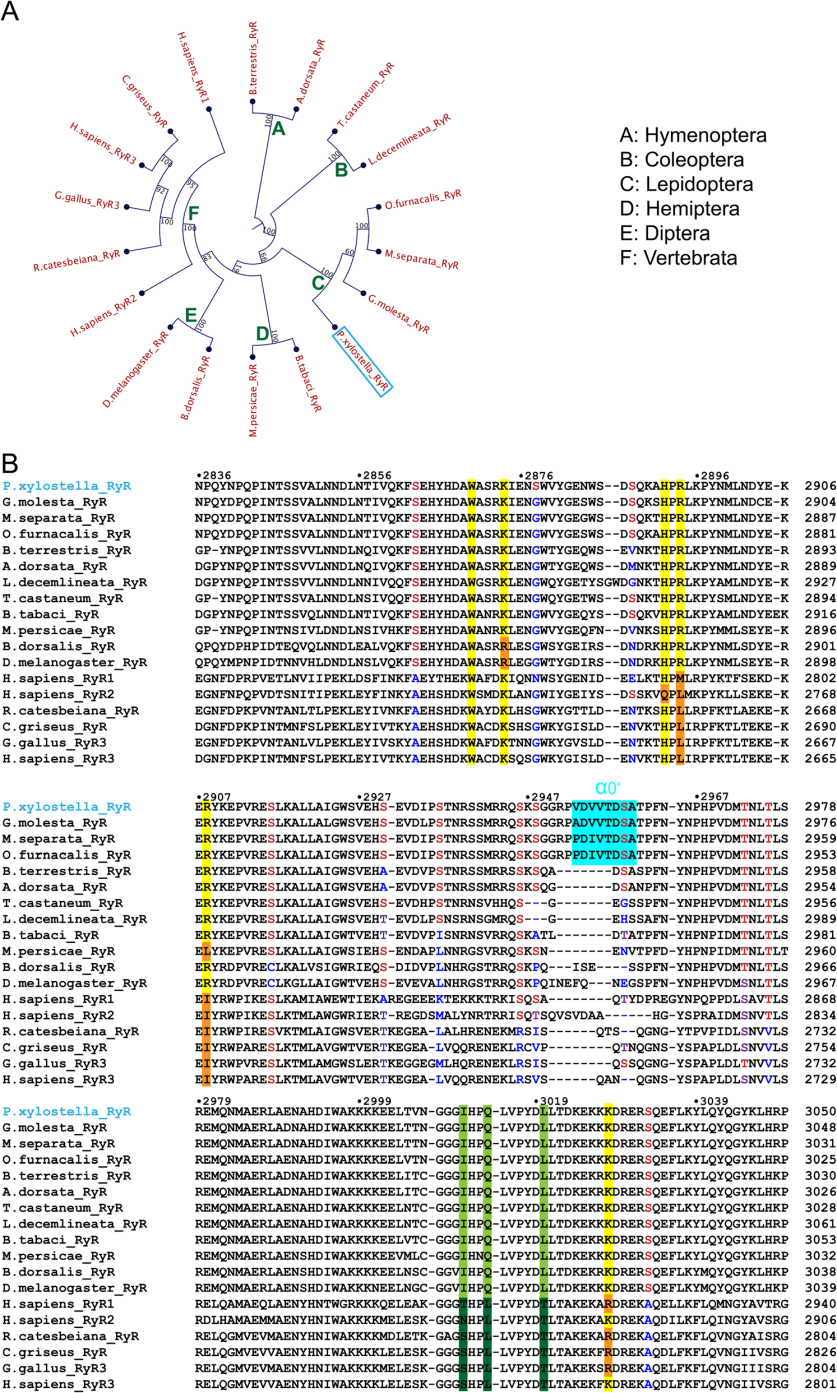
Phylogenetic analysis and sequence alignment. **a** Phylogenetic analysis of RyRs from different species, including Lepidoptera (*Plutella xylostella*: NP_001296002.1, *Grapholitha molesta*: ALM96708.1, *Mythimna separate*: AWV67093.1, *Ostrinia furnacalis*: AGH68757.1), Hymenoptera (*Bombus terrestris*: XP_012175583.1, *Apis dorsata*: XP_006622367.1), Coleptera (*Leptinotarsa decemlineata*: AHW99830.1, *Tribolium castaneum*: NP_001308588.1), Hemiptera (*Bemisia tabaci*: AFK84957.1, *Myzus persicae*: XP_022160123.1), Diptera (*Bactrocera dorsalis*: AHY02115.1, *Drosophila melanogaster*: NP_001246211.1), and vertebrate (*Homo sapiens*1: NP_000531.2, *Homo sapiens*2: NP_001026.2, *Rana catesbeiana*: BAA04647.2, Cricetulus griseus: ERE72086.1, *Gallus gallus*: NP_996757.2, *Homo sapiens*3: NP_001027.3). **b** Sequence alignment of RyRs from different species (same as in panel A). The phosphorylation sites are colored in red (conserved), purple (semi-conserved), or blue (non-conserved); the glycerol-coordinating residues are highlighted in yellow (conserved) or orange (non-conserved); the insect-specific HD1-interacting residues are highlighted in light green (conserved) or dark green (non-conserved); the Lepidoptera-specific helix α0′ is highlighted in cyan

We created a phylogenetic tree based on the sequences of RyRs from select 12 insects and six vertebrate species (Fig. 9a). According to the tree, RyR of DBM, a representative species in Lepidoptera, has similar phylogenetic distance to both Hemiptera and Coleoptera, which agrees with the species-specific toxicology data of diamide insecticides [62]. Both Hymenoptera and Coleoptera branched earlier than Lepidoptera. Within vertebrates, mammalian RyR2 is generally closer to insect RyRs compared to the other two isoforms.

The sequence alignment shows four important aspects. First, the unique helix α0′ containing two phosphorylation sites located in DBM phosphorylation loop only exists in Lepidoptera, including *Plutella xylostella* (NP_001296002.1), *Spodoptera exigua* (ALL55466.1), *Spodoptera litura* (XP_022828487.1), *Grapholitha molesta* (ALM96708.1), *Cnaphalocrocis medinalis* (AFI80904.1), *Mythimna separate* (AWV67093.1), *Galleria mellonella* (XP_026764893.1), and *Ostrinia furnacalis* (AGH68757.1), suggesting that this special phosphorylation regulation pattern is Lepidoptera-specific (Fig. 9b). Second, the phosphorylation sites we identified by LC-MS can be classified into three groups according to their conservativity: (1) S2878 is only present in DBM; (2) S2863, S2936, S2959, and S3033 are the most conserved in insects; and (3) S2888, S2916, S2930, S2946, S2948, T2973, and T2976 are found both in insects and vertebrates. Third, three residues (Ile3011, Gln3014, and Leu3020) in the HD1-interacting interface of Repeat34 are only conserved in insects (Fig. 9b). Fourth, the glycerol-binding pocket identified in DBM Repeat34 is conserved in all insects but not in vertebrates. Two key arginine residues (Arg2894 and Arg2908) in the pocket are replaced by neutral hydrophobic residues in vertebrate RyRs (Fig. 9b).

## Discussion

RyR has been used as a major insecticide target since the late 2000s. Recent emergence of resistance to all three major diamide insecticides in many destructive agricultural pests is threatening the efficacy of these crucial crop-protection tools. There is a pressing need to develop novel pesticides with different mode of actions. In this study, we investigated the phosphorylation of DBM RyR with the aim to provide the molecular basis for the regulation of this important insecticide target protein.

By tandem mass spectrometry, we have identified 12 PKA phosphorylation sites within the Repeat34 domain of DBM RyR, which makes this domain a phosphorylation hotspot. Interestingly, the phosphorylation specificity depends on the temperature: at a lower temperature, fewer sites are phosphorylated. To gain more insights into the molecular mechanism of this phosphorylation regulation, we have solved the crystal structure of this domain at 1.85 Å resolution. The structure shows that most phosphorylation sites cluster in a region containing a loop and a short α helix (Fig. 6). By bioinformatics analysis, we have found that this short α helix only exists in Lepidoptera, suggesting some species-specific regulation (Fig. 9b). Typically, PKA binds its substrate in an unfolded state, such as in the cases of phospholamban and mammalian RyR1 and RyR2. Thus, we suspected this peculiar α helix is more dynamic and not thermally stable. As we predicted, the thermal melt assay revealed that the melting temperature of DBM Repeat34 was only 35 °C, which was 13 °C lower than that of mammalian RyRs (Fig. 5). The low thermal stability might be due to the evolutional adaptation to the lower environmental temperature of insects. It also suggested that at a higher reaction temperature (30 °C), a significant portion of the domain is unfolded and becomes more accessible to PKA. By contrast, at a lower reaction temperature (18 °C), the domain is fully folded and only the flexible regions with high dynamics could be phosphorylated by PKA. The observation that the temperature-dependent phosphorylation sites are close to the constitutive phosphorylation sites in the three-dimensional structure suggests that they might have additive effect, which is used to fine-tune the activity of the channel. Many insects have a wide range of ambient temperature, so this temperature-dependent phosphorylation regulation might have important physiological function for them to adapt to the environment. We detected fewer phosphorylation sites in the full-length insect RyR, suggesting that the phosphorylation might be kept at a lower basal level without the activation of PKA.

We have also solved the crystal structure of the phospho-mimetic mutant S2946D of DBM Repeat34. The mutation is located in a flexible loop region and does not significantly change the structure of the domain (Fig. 1e). By docking the domain into the cryo-EM model of full-length RyR2, we found that Repeat34 is located at the top turret region of RyR cytoplasmic region, and Ser2946 and most other phosphorylation sites are in a region protruding away from the main body of the channel, which is structurally less conserved in Repeat34 (Fig. 7). During EC-coupling, RyR is activated by voltage-gated calcium channel (CaV) located in the plasma membrane through the physical interaction between these two channels [63]. The location of Repeat34 and its phosphorylation sites within the full-length RyR implies that this domain might play an important role in interacting with the neighboring SPRY3 domain and that phosphorylation might regulate this process. Previous studies also showed that phosphorylation might affect the binding of an important regulator called FKBP12, although contradictory evidence has made this claim controversial [36, 64, 65]. The relationship between phosphorylation regulation and FKBP-binding in insects remains to be explored.

Among all the structural differences between DBM and mammalian Repeat34 domains, a unique glycerol-binding pocket has particularly drawn our attention (Fig. 4). The movement of helix α1, helix α2, and loop α1-α2 towards helix α2′ forms this new pocket in DBM Repeat34. It is mainly a positively charged surrounding composed of one histidine, two arginine, and two lysine residues. The binding of glycerol, which was conformed by ITC, might have induced the large structural difference we observed between DBM and mammalian Repeat34 domains, hinting at its potential function in modulating the structure and activity of DBM RyR. Drugs using glycerol as scaffold [66] and drug pockets rich in positive charges [67, 68] have both been reported in previous studies. Bioinformatics analysis shows that this pocket is conserved only in insects, which makes it a potential target for designing insect-specific green pesticides with the potential to fight the current resistance crisis.

## Conclusions

In conclusion, insect RyR has a different phosphorylation regulation compared to mammalian RyRs. First, DBM Repeat34 domain has an additional α-helix near the phosphorylation loop, where several newly identified PKA sites cluster. Second, DBM RyR’s phosphorylation is temperature-dependent, facilitated by the dynamic structure of the domain. Third, we identify an insect-specific glycerol-binding pocket in DBM Repeat34. Its druggability and potential application in design selective insecticides still needs to be further explored.

## Methods

### Protein cloning, expression, and purification

DBM RyR residues 2836–3050 (GENBANK accession NM_001309073) and mouse RyR2 residues 2699–2904 (GENBANK accession NM_001309073) were cloned into the pET28HMT vector, which contains an N-terminal His-tag, an MBP-tag, and a TEV cleavage site [69]. Surface entropy reduction and phosphor-mimetic mutations were introduced via Quickchange (TianGen). *Escherichia coli* BL21 (DE3) cells (NEB) were transformed with the plasmid and plated on LB agar supplemented with 50 μg/ml kanamycin. The cells were grown at 37 °C in 2YT medium supplemented with 50 μg/ml kanamycin. When OD_600_ reached ~ 0.8, the cells were induced with 0.5 mM β-D-1-thiogalactopyranoside (IPTG) and grown at 18 °C for 16 h prior to harvesting by centrifugation (8000×*g* for 10 min at 4 °C).

The cells were disrupted via sonication in lysis buffer (10 mM HEPES pH 7.4, 250 mM KCl, 0.02 mg/ml DNase I, 0.2 mg/ml lysozyme, 1 mM phenylmethanesulfonyl fluoride). The cell debris was removed by centrifugation (20,000×*g* for 10 min at 4 °C). The soluble fraction was filtered through a 0.22-μm filter and loaded onto a 10-ml HisTrap HP column (GE Healthcare) pre-equilibrated with buffer A (10 mM HEPES pH 7.4, 250 mM KCl, 5 mM β-mercaptoethanol). The column was eluted using a linear gradient of 20–250 mM imidazole in the elution buffer. The elute was dialyzed against 2 L buffer A for 4 h to remove imidazole and then loaded on an amylose resin column (New England Biolabs). The collected protein was cleaved with recombinant TEV protease (1:70 M ratio) during overnight dialysis against 2 L buffer A. The dialyzed sample was loaded on another HisTrap HP column (GE Healthcare) to remove the His-MBP-tag. The flow-through was collected and dialyzed against 2 L buffer B (50 mM Tris–HCl, pH 6.8, 5 mM β-mercaptoethanol) for 4 h. RyR protein was further purified by a SP Sepharose high-performance column (GE Healthcare) using a linear gradient from 20 to 500 mM KCl in elution buffer (10 mM Tris pH 6.8, 5 mM β-mercaptoethanol). Finally, the protein was concentrated using Amicon concentrators (10 K MWCO from Millipore) and injected on a Superdex 200 26/600 gel-filtration column (GE Healthcare) and eluted with buffer A. The protein purity was examined on a 15% (w/v) SDS polyacrylamide gel. The protein sample was concentrated to 8 mg/ml and exchanged to the crystallization buffer (10 mM HEPES pH 7.4, 50 mM KCl, 1 mM tris (2-carboxyethyl) phosphine) before stored at − 80 °C. The purification of sarcoplasmic reticulum (SR) of *Bombyx mori* (silkworm) was performed as described before [70].

### Crystallization, data collection, and structure determination

Surface entropy reduction mutations D3023A, K3024A, and K3027A were introduced to DBM RyR Repeat34 domain to facilitate the crystallization process. Protein crystals were grown using the hanging-drop method at 20 °C. The crystals of wild type (8 mg/ml) were grown in 1.8 M ammonium citrate tribasic (pH 7.0), which was transferred to the same solution supplemented with 30% glycerol before flash cooling in liquid nitrogen. The crystals of S2946D mutant (5 mg/ml) were grown in 2% (v/v) PEG400, 0.1 M HEPES (pH 7.5), and 2.0 M ammonium sulfate, and flash cooled using the same solution supplemented with 30% glycerol as cryo-protectant.

Diffraction data from single crystals were collected on BL18U1 at Shanghai Synchrotron Radiation Facility (SSRF) to a resolution of 1.85 Å and 1.53 Å for wild type and S2946D, respectively. The datasets were indexed, integrated, and scaled using HKL3000 suite. Molecular replacements were performed using PHENIX [71]. The first half of RyR1 Repeat12 (861–936) and the second half of RyR2 Repeat34 (2820–2904) were used as two separate search models for wild-type DBM Repeat34. After running Phaser-MR, we replaced the model sequence with the object sequence. We then used the wild type as a search model to solve the phase problem of S2946D. The structure was further manually built into the modified experimental electron density using Coot [72] and refined in PHENIX [71] in iterative cycles. The data collection and final refinement statistics are shown in Table 1. All structure figures were generated using UCSF Chimera [56].

### Fluorescence-based thermal shift assays

The protein melting curves were measured using fluorescence-based thermal shift assays [73]. Ten microliters of 3 mg/mL protein was mixed into a 500-μl solution containing 2 × Sypro orange dye (Sigma). The temperature of the solution was slowly increased from 25 °C to 95 °C with 0.033 °C/s steps in a QuantStudio 6 Flex real-time PCR machine (Life, CA, U.S.A.). The melting curves were plotted according to the fluorescence signal change during the heating. Melting temperatures were obtained by taking the transition midpoints.

### Isothermal titration calorimetry

The purified DBM RyR Repeat34 and RyR2 Repeat34 proteins were dialyzed against 150 mM KCl, 10 mM HEPES (pH 7.4), 2 mM TCEP at 4 °C. The glycerol was dissolved in the same solution to concentration of 200 μM. Titrations consisted of 20 injections of 2 μL of 200 μM glycerol into the cell solution containing 25 μM Repeat34 or control buffer. The reference cell was filled with water. Experiments were performed at 4 °C and a stirring speed of 750 rpm on a PEAQ-ITC instrument (Malvern).

### In vitro phosphorylation

Phosphorylation reactions were performed at 30 °C or 18 °C overnight in 50-μl volumes. The reaction mixture for PKA contained 10 mM HEPES (pH 7.4), 50 mM KCl, 20 mM MgCl_2_, 0.2 mM ATP, 2 mM 2-mercaptoethanol, 0.8 mg/ml DBM RyR Repeat34, and 200 U PKA (Creative Biomart). The unit of PKA is defined as the amount of enzyme required to incorporate 1 pmol of phosphate into casein in 1 min. DBM PKA and human PKA share a relatively high sequence identity and a conserved activity center, so we chose to use the commercial human PKA for this assay.

Phosphorylated proteins were alkylated with 55 mM iodoacetamide. Peptides were separated by a 120-min gradient elution at a flow rate of 0.30 μL/min with a Thermo EASY-nLC 1200 system, which was directly interfaced with Orbitrap Fusion Lumos Tribrid mass spectrometer. The analytical column (100 μm ID, 150 mm length) packed with Reprosil-Pur 120 C18-AQ beads (Dr. Maisch) was a fused silica capillary column with spray tip. Mobile phase A consisted of 0.1% formic acid, and mobile phase B consisted of 80% acetonitrile and 0.1% formic acid. Gradients were run from 8 to 12% B over 10 min, 12 to 27% over 69 min, 27 to 45% over 28 min, 45 to 95% over 3 min, and 95% over 10 min. The mass spectrometer was operated in the data-dependent acquisition mode using Xcalibur 4.0 software, and there was a single full-scan mass spectrum in the Orbitrap (350–1800 m/z, 120,000 resolution) followed by data-dependent MS2 scans at 35% collision energy (HCD) in an ion trap.

MS/MS spectra from each LC-MS/MS run were searched against the DBM proteins using Proteome Discoverer (Version 2.2) searching algorithm. The search criteria were as follows: full tryptic specificity was required; two missed cleavages were allowed; carbamidomethylation (C) was set as fixed modification; oxidation (M) and Phospho (S, T, Y) were set as dynamic modifications; precursor ion mass tolerance was 20 ppm for all MS acquired in the Orbitrap mass analyzer; and fragment ion mass tolerance was 0.6 Da for all MS2 spectra acquired in the ion trap. High confidence score filter (FDR < 1%) was used to select the “hit” peptides and their corresponding MS/MS spectra were manually inspected.

## Data Availability

The atomic coordinates and structure factors for DBM RyR Repeat34 (PDB ID 6J6O) and DBM RyR Repeat34 S2946D (PDB ID 6J6P) have been deposited in the RCSB Protein Data Bank.

## Abbreviations

RyRs: Ryanodine receptors
DBM: Diamondback moth
PKA: Protein kinase A
EC-coupling: Excitation-contraction coupling
Cryo-EM: Cryo-electron microscopy
ASU: Asymmetric unit
RMSD: Root mean square deviation
CaMK II: Calmodulin-dependent kinase II
Cav: Voltage-gated calcium channel
CPVT: Catecholaminergic polymorphic ventricular tachycardia
ARVD: Arrhythmogenic right ventricular dysplasia
MH: Malignant hyperthermia
CCD: Central core disease
AD: Alzheimer’s disease
AKH1: Adipokinetic hormone 1
AKHR: Adipokinetic hormone receptor
ErGPCR: Ecdysone responsive G-protein-coupled receptors
CREB: cAMP response element-binding protein
Lspdp1: Lipid storage droplet protein 1
BR-C: Broad-Complex transcription factor
IPTG: β-D-1-Thiogalactopyranoside
TCEP: Tris(2-carboxyethyl)phosphine
SSRF: Shanghai Synchrotron Radiation Facility
HCD: High-energy C-trap dissociation
FDR: False discovery rate
